# Prognostic Relevance and In Vitro Targeting of Concomitant PTEN and p16 Deficiency in Chordomas

**DOI:** 10.3390/cancers15071977

**Published:** 2023-03-26

**Authors:** Carolin Seeling, Elena Mosca, Eva Mantel, Peter Möller, Thomas F. E. Barth, Kevin Mellert

**Affiliations:** 1 Institute of Pathology, University Hospital of Ulm, 89081 Ulm, Germanykevin.mellert@uni-ulm.de (K.M.); 2 Department of Internal Medicine III, University Hospital Ulm, 89081 Ulm, Germany

**Keywords:** chordoma, PTEN, PI3K/AKT/mTOR signaling, *CDKN2A*, p16

## Abstract

**Simple Summary:**

Chordomas are rare malignant bone tumors that predominantly occur along the spine. The most frequent alterations include the loss of PTEN and p16 proteins. While both oncoproteins have been investigated as independent prognostic factors, in our chordoma cohort of 43 patients we were able to define a subgroup of 16% harboring the concomitant loss of PTEN and p16. We could show that these patients had a shorter overall survival. We exploited the oncogenic driver addiction in these chordomas by simultaneously inhibiting the PI3k/AKT/mTOR and CDK4/6 pathways. We observed synergistic effects for the drug combination of rapamycin and palbociclib, providing a promising novel strategy, especially for PTEN^−^/p16^−^ chordoma patients.

**Abstract:**

Chordomas are rare bone tumors arising along the spine. Due to high resistance towards chemotherapy, surgical resection—often followed by radiation therapy—is currently the gold standard of treatment. So far, targeted systemic therapies have not been approved. The most frequent molecular alterations include the loss of *PTEN* and *CDKN2A* (encoding p16), being associated with poor prognoses in chordoma patients. Specific inhibitors of the PI3K/AKT/mTOR pathway as well as CDK4/6 have shown antitumor activity in preclinical studies and have recently been under investigation in phase II clinical trials; however, the clinical impacts and therapeutic consequences of concomitant PTEN and p16 deficiency have not yet been investigated in chordomas. In a cohort of 43 chordoma patients, 16% of the cases were immunohistochemically negative for both markers. The simultaneous loss of PTEN and p16 was associated with a higher KI-67 index, a tendency to metastasize, and significantly shorter overall survival. Additionally, 30% of chordoma cell lines (*n* = 19) were PTEN-/p16-negative. Treating these chordoma cells with palbociclib (CDK4/6 inhibitor), rapamycin (mTOR inhibitor) or the pan-PI3K inhibitor buparlisib significantly reduced cell viability. Synergistic effects were observed when combining palbociclib with rapamycin. In conclusion, we show that patients with PTEN-/p16-negative chordomas have poor prognoses and provide strong preclinical evidence that these patients might benefit from a Palbociclib/rapamycin combination treatment.

## 1. Introduction

Chordomas are rare, slowly but locally aggressive growing tumors believed to originate from residual notochordal cells [1]. Correspondingly, chordomas usually arise within the bones of the axial skeleton and express high levels of the embryonic transcription factor brachyury [2]. *En bloc* resection is the recommended treatment whenever feasible; however, due to the profound proximity of chordomas to critical nerval structures, such as the spinal cord and brain stem, surgical excision often remains incomplete [3]. Therefore, in recent years many research efforts have been devoted to the identification and systemic therapeutic inhibition of molecular chordoma drivers. *CDKN2A* copy number loss has been identified as the most frequent genomic alteration in chordomas, resulting in the absence of the p16INK4a protein, an inherent negative regulator of cyclin-dependent kinases 4 and 6 (CDK4/6) [4,5]. Conclusively, the CDK4/6 inhibitor palbociclib (Ibrance^®^) has been shown to reduce the proliferation of various chordoma cell lines [4]. A phase II clinical trial has recently been examining the effectivity of palbociclib as a single-agent treatment in chordoma patients (NCT03110744). Nevertheless, little is known about the prognostic relevance of p16 negativity, and reports on its possible impact on survival are inconclusive [6,7,8].

In parallel, alterations in the phosphatidylinositol 3-kinase (PI3K) signaling genes have frequently been reported in multiple chordoma studies. Among them, copy number loss at the *PTEN* gene locus on chromosome 10q23.3 is the most common genomic alteration in chordomas, affecting PI3K/AKT/mTOR signaling [8,9,10,11]. PTEN is a phosphatase that dephosphorylates cytoplasmatic PIP3 and thereby directly opposes the activation of the oncogenic PI3K/AKT/mTOR signaling network. As a tumor suppressor, PTEN is involved in various biological processes, such as cell metabolism, motility, and proliferation. Nuclear PTEN plays a role in DNA repair and centromere stability (reviewed in [12]). The therapeutical experience with inhibitors of the PI3K/AKT/mTOR pathway in chordomas is restricted to single case reports and clinical trials with small cohorts. The mTOR inhibitor rapamycin is among the most frequently applied small molecules, with heterogeneous outcomes. Additionally, the first preclinical experiments with the novel pan-PI3K inhibitor buparlisib have provided convincing results in chordoma xenograft models [13]. Recently, PTEN has been recognized as a prognostic factor in chordomas, and the loss of PTEN was correlated with a shorter progression-free survival (PFS) as well as faster proliferation [8,14].

The frequency and prognostic impact of the coincidental loss of p16 and PTEN in chordomas have not been assessed. Here, we address the prognostic relevance and oncogenic function of PTEN with regard to CDK4/6 signaling in chordoma tissue samples and derived cell lines. We investigated the effectivity of the pan-PI3K inhibitor buparlisib and mTOR inhibitor rapamycin as single agents, as well as in combination with the CDK4/6 inhibitor palbociclib, in chordoma cell lines. We provide preclinical evidence and proof of concept experiments demonstrating that the combined inhibition of the two most frequently altered key signaling pathways is a promising strategy for chordoma treatment.

## 2. Materials and Methods

### 2.1. Cell Culture

**Figure 1:**
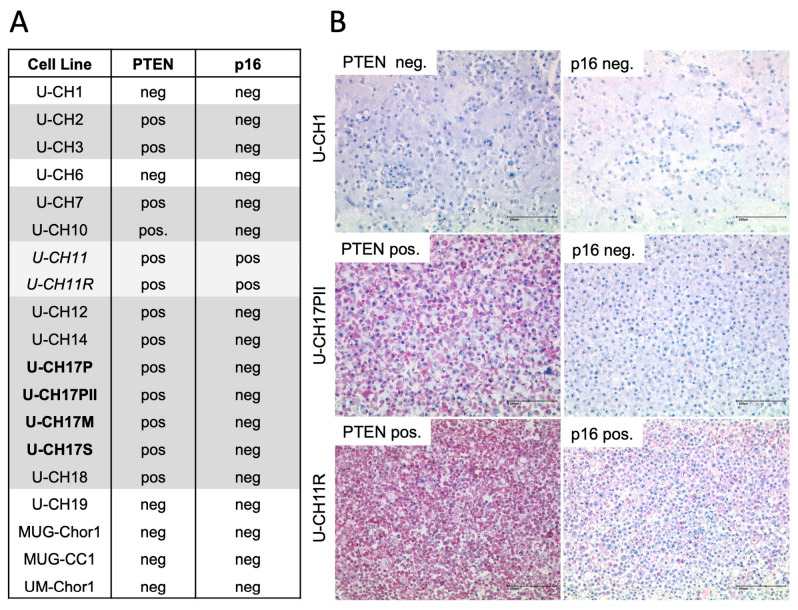
Immunocytochemical analysis of PTEN and p16 in chordoma cell lines. (**A**) Overview of the included cell lines with the respective PTEN and p16 statuses. (**B**) Immunocytochemical staining of PTEN and p16 in chordoma cell lines (scale bar = 200 µm). U-CH1 is PTEN- and p16-negative, whereas U-CH17PII is PTEN-negative and p16-positive. U-CH11R is positive for PTEN and p16.

Nineteen chordoma cell lines (see Figure 1A) derived from fifteen different patients were included. Cells were cultured in Iscove’s Modified Dulbecco’s Medium/RPMI 1640 (4:1, Lonza, Basel, Switzerland) with 10% fetal bovine serum (Biochrom AG, Berlin, Germany), 2 mM glutamine, and 1% penicillin/streptomycin (both Lonza, Basel, Switzerland), and maintained at 37 °C in a 5% carbon dioxide (CO_2_) humidified incubator (Thermo Fischer Scientific, Waltham, MA, USA). The quality control of cell cultures was performed via short tandem repeat (STR) analyses and mycoplasma contamination screenings.

### 2.2. Chordoma Tissue Bank and Survival Analysis

Chordoma samples from 43 patients of the Ulm tissue bank (mean age of diagnosis: 61.02 years; range: 17–84 years; and 28 males, 15 females) were analyzed with regard to associations between immunohistochemical PTEN as well as p16 status and clinicopathological features (gender, age, KI-67 index, maximal diameter, recurrence, metastasis, and location). Statistic testing was performed by a chi-squared test, Student’s *t*-test, and Fisher’s exact test.

The survival statuses for 40 patients were available, with a mean follow-up time of 6.91 ± 6.37 years. Overall survival (OS) was defined as the interval between the first diagnosis and the date of death from any cause. Survival curves were drawn using the Kaplan–Meier method and compared via the logrank (Mantel–Cox) test. The median survival (MS) and the hazard (HR) were calculated using GraphPad Prism (Graphpad Software Inc., San Diego, CA, USA).

### 2.3. Immunohistochemistry

Immunohistochemical staining was performed through the use of the avidin–biotin complex (ABC) method, applying the K005 AP/RED detection system (Dako, Glostrup, Denmark) on 4 µm-thick sections of formalin-fixed and paraffin-embedded cell as well as tissue blocks.

The primary antibodies used were rabbit monoclonal anti-PTEN (138G6, Cell Signaling, Danvers, MA, USA, 1:100) and p16INK4a (1D7D2, Invitrogen, Carlsbad, CA, USA, 1:400). Immunostainings were evaluated by an experienced pathologist who was blinded to the clinicopathological parameters and clinical outcomes of the patients.

### 2.4. Protein Isolation and Western Blot Analyses

The cells were rinsed twice with ice-cold PBS and then lysed in a RIPA buffer containing a protease and phosphatase inhibitor cocktail (Sigma-Aldrich, St. Louis, MO, USA). The protein lysates were purified by centrifugation at 14,000 rpm at 4 °C for 15 min. The total protein and an appropriate weight marker (Chamelon^®^ Vue Pre-stained Protein Ladder, LI-COR Biosciences, Lincoln, NE, USA) were separated using SDS-PAGE and transferred to nitrocellulose membranes by applying a wet tank blot transfer system. Membranes were blocked with 5% skim milk for 60 min, following the incubation of the primary antibodies at 4 °C overnight. The incubation of the appropriate secondary antibodies was performed for 60 min at room temperature. Finally, the detection of the proteins was carried out by using the WesternSure PREMIUM Chemiluminescent Substrate and a C-DiGit Blot Scanner (LI-COR Biosciences).

The following antibodies were used: PTEN (138G6; Cell Signaling, Danvers, MA, USA, 1:1000), AKT (polyclonal; Cell Signaling, 1:1000), phospho-AKT (Ser473; D93; Cell Signaling, 1:1000), S6 ribosomal protein (5G10; Cell Signaling), phospho-S6 ribosomal protein (Ser240/244; D68F8; Cell Signaling; 1:1000), histone H3 (D1H2; Cell Signaling; 1:2000), alpha-tubulin (DM1A; Cell Signaling; 1:1000), anti-rabbit and anti-mouse IgG antibodies produced in goat (Sigma Aldrich, St. Louis, MO, USA).

### 2.5. MTS Cell Viability Assay

Cells were seeded at a density of 7500 cells/cm^2^ in 96-well flat-bottom culture plates and allowed to adhere for 24 h at 37 °C. Subsequently, cells were subjected to treatment with single or combined inhibitors (buparlisib, rapamycin, palbociclib), DMSO, or H_2_O (vehicle controls) for 6 days. Cell viability was assessed via MTS assays (Abcam, Cambridge, UK) according to the manufacturer’s recommendations. The absorbance (490 nm) was measured with a multiplate spectrometer (Epoch, Bioteck, Bad Friedrichshall, Germany). IC50 values were determined using the GraphPad Prism Software (GraphPad Software, Inc.). For combination treatments, constant-ratio dose series based on individual IC50 values of palbociclib and rapamycin, alongside buparlisib, were used. All of the cell lines were tested in biological and technical triplicates.

Inhibitors were obtained from Selleckchem (Houston, TX, USA) and dissolved in DMSO or H_2_O according to the manufacturer’s recommendation. Buparlisib (BKM120) is a selective PI3K inhibitor of p110α/β/δ/γ, rapamycin (sirolimus) is a specific mTOR inhibitor, and palbociclib HCl (PD-0332991) is a highly selective inhibitor of CDK4/6.

### 2.6. Isobologram Analysis of Drug Interactions

For the quantitative assessment of synergism, the CI-isobol method of Chou–Talalay was used [15]. A combination index (CI) was computed from the dose–effect data of single- (Supplementary Figure S1) and combined-drug treatments. A CI < 1 indicates synergism; a CI = 1 indicates additive effect; and a CI > 1 indicates antagonism. The results were summarized as normalized isobolograms. The effect of a drug treatment is depicted as a point in the isobologram.

## 3. Results

### 3.1. PTEN and p16 Deficiency in Chordoma Cell Lines

PTEN and p16 protein levels in 19 chordoma cell lines were assessed via immunocytochemistry (Figure 1). Tumor progression models U-CH11 and U-CH11R, as well as U-CH17PII, U-CH17M, and U-CH17S, were derived from the same patient. Negative PTEN staining was observed in 6/19 (31.6%) of the cell lines, including U-CH1, U-CH6, U-CH19, MUG-Chor1, MUG-CC1, and UM-Chor1. All of the cell lines, except U-CH11 and U-CH11R, were negative for p16 upon immunocytochemical staining (17/19, 89.5%). All PTEN-negative cell lines depicted the concomitant loss of p16 (6/19, 31.6%), whereas the singular loss of p16 was observed in 11/19 (57.9%) of the cell lines. U-CH11 and U-CH11R were neither negative for PTEN nor for p16. In summary, the concomitant loss of p16 and PTEN is a frequent event in chordoma cell lines.

### 3.2. Concomitant Loss of Both PTEN and p16 Predicts a Shorter Overall Survival in Chordoma Patients

**Figure 2:**
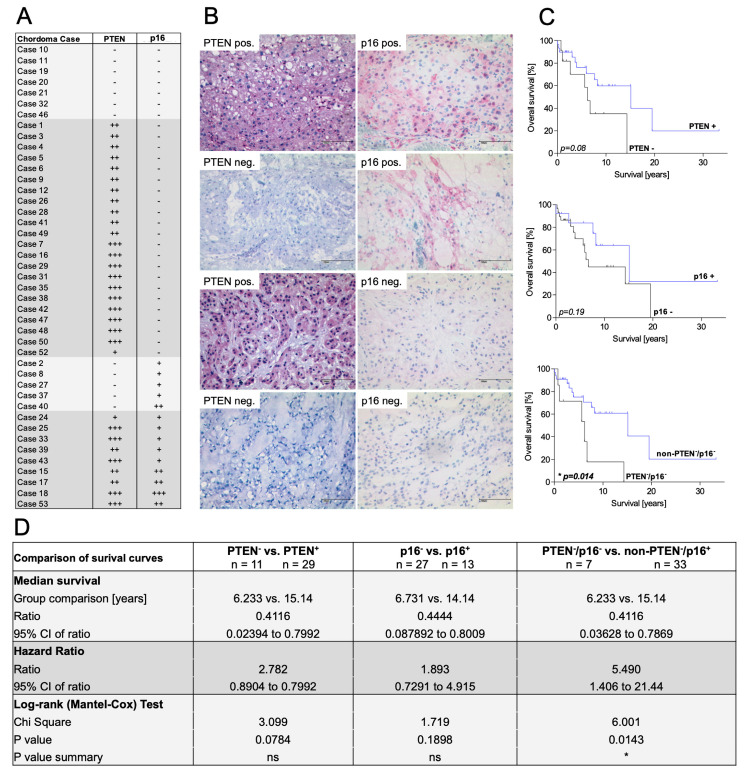
Immunohistochemical analysis of the chordoma tissue cohort. (**A**) Overview of included chordoma patients and their respective PTEN and p16 statuses. Arbitrary scoring -, +, ++, +++ was used to assess the degree of immunostaining. (**B**) Different constellations of immunohistological PTEN and p16 staining in selected chordoma tissues (scale bar = 100 µm). (**C**) Overall survival in terms of dependence on the PTEN and p16 statuses. A significantly shorter overall survival was observed in PTEN^−^/p16^−^ cases. (**D**) Median survival, hazard ratio, and logrank (Mantel–Cox) test of the subgroups.

In our chordoma tissue cohort, 29/43 (67%) of the patients presented immunohistochemical negativity for the p16 protein (Figure 2A,B). PTEN deficiency was observed in 12/43 (28%); the simultaneous absence of both markers was found in 7/43 (16%). Therefore, the previously described common loss of p16 and PTEN within the chordoma cell lines was confirmed in the tissue cohort.

We evaluated if the singular loss of p16 and PTEN or the combined loss of both markers correlates to clinical aspects in our cohort. No significant differences in gender, age at first diagnosis, KI-67 index, and tumor location, size, and tendency to recur or metastasize were observed with regard to p16-positive (*n* = 14) and p16-negative (*n* = 29) chordoma samples (Table 1). Comparing PTEN-positive (*n* = 31) and PTEN-negative (*n* = 12) samples, significant differences were found regarding age at first diagnosis (*p* = 0.049), proliferation speed (KI-67 index, *p* = 0.04), and the tendency to metastasize (*p* = 0.0026). Differences in KI-67 indices (*p* = 0.04) and metastatic rates (*p* = 0.0026) persisted when comparing PTEN^-^/p16^-^ cases (*n* = 7) to cases with only one or no negative marker (non-PTEN^−^/p16^−^; *n* = 36).

As fast tumor proliferation and high rates of metastasis may contribute to shorter survival, we performed Kaplan–Meier overall survival analyses. Three patients had to be excluded due to missing follow-up data.

Neither the singular loss of PTEN nor the singular loss of p16 significantly affected overall survival (Figure 2C); however, the subgroup of PTEN^−^/p16^−^ patients (*n* = 7) had a markedly worse prognosis than the control group. The overall survival of PTEN^−^/p16^−^ patients was 6.2 years, whereas it was more than 15 years in the control group (*p* = 0.014; hazard ratio ~5.5; Figure 2D). Based on these data, we defined a novel subgroup of PTEN^−^/p16^−^ chordoma patients with unfavorable clinical courses.

### 3.3. Combined Inhibition of the PTEN and p16 Signaling Pathways

Due to the clinical relevance of concomitant PTEN and p16 deficiency in chordomas, we aimed to establish treatments targeting the altered pathways. The small molecule palbociclib was used to restore the missing function of the cell cycle regulator p16, which resulted from the genomic loss of the *CDKN2A* gene locus. Buparlisib is a novel, orally administrable pan-class I PI3K inhibitor, which is currently under investigation in a range of solid tumors as well as lymphoid malignancies, replacing the inhibitory function of PTEN in PIP2 to PIP3 conversion. Rapamycin is an FDA-approved inhibitor of mTORC1, a key complex downstream of PI3K/AKT-signaling, resulting in the blockage of cell cycle progression.

Combination treatments were tested in six chordoma cell lines, four of which harbor concomitant p16 and PTEN loss, namely U-CH1, MUG-CC1, MUG-Chor1, and UM-Chor1. The cell lines U-CH2 and U-CH17PII are p16-negative but positive for PTEN in the immunocytochemical analyses.

**Figure 3:**
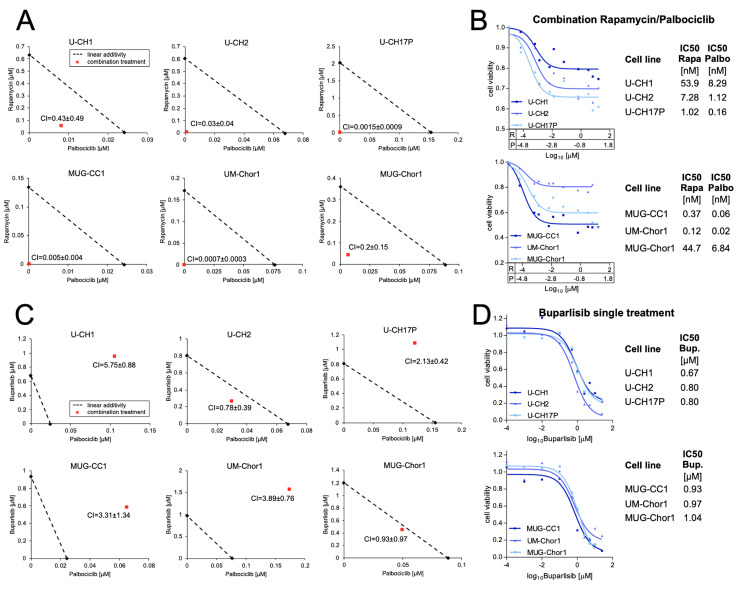
Targeting CDK4/6 and PI3K/AKT/mTOR signaling in chordoma cell lines. Isobolograms of the treatments with palbociclib in combination with rapamycin (**A**). Inhibition curves and the corresponding IC50 values of the combination treatment with rapamycin (R/Rapa) and palbociclib (P/Palbo) (**B**). Isobolograms of palbociclib combined with buparlisib (**C**) and inhibition curves of buparlisib monotherapy as well as corresponding IC50 values (**D**) in six chordoma cell lines.

The Chou–Talalay method was applied to determine the combination index of two different inhibitors. The resulting combination indices (CIs) indicated no synergistic effect for the combination of buparlisib and palbociclib (Figure 3C); however, singular inhibition with buparlisib resulted in a dramatic reduction in cell viability to less than 20% in all tested cell lines, with IC50 values ranging from 0.7 µM ± 0.11 µM in cell line U-CH1 to 1.0 µM ± 0.46 µM in cell line MUG-Chor1, suggesting that buparlisib can serve as a promising monotherapy in chordoma patients (Figure 3D). Though the above may be the case, no difference between PTEN-negative (*n* = 4) and PTEN-positive (*n* = 2) cell lines was observed (*p* = 0.41). In all six cell lines tested, a synergistic effect of rapamycin (R) and palbociclib (P) was observed (Figure 3A). The resulting inhibition curves of the combinations are given in Figure 3B, showing a clear reduction in cell viability in all of the cell lines, with IC50 values ranging from 0.12 nM (R) and 0.02 nM (P) in chordoma cell line UM-Chor1 to 53.9 nM (R) and 8.29 nM (P) in U-CH1. Independent of the PTEN status, the growth inhibitory effects were comparable in all of the tested cell lines (*p* = 0.39). Singular inhibition using rapamycin or palbociclib also resulted in reduced cell viability; however, markedly higher concentrations of the compounds were required to induce similar viability reductions (Supplementary Figure S1).

**Figure 4:**
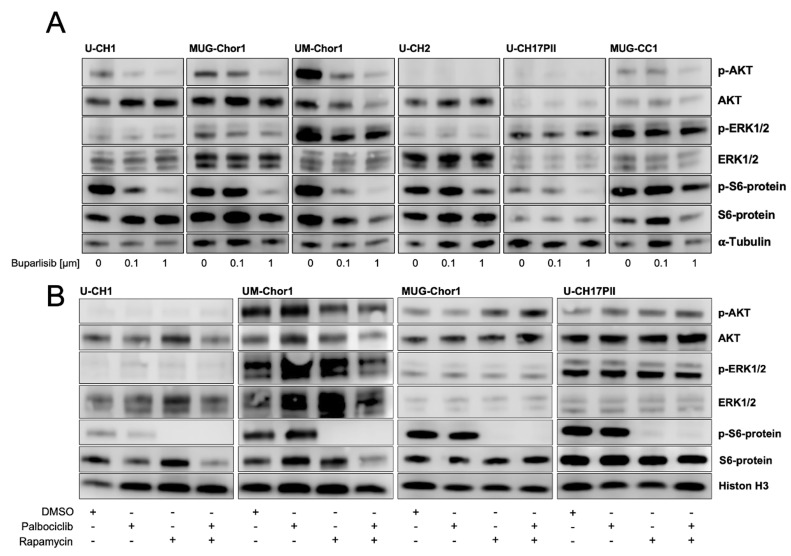
Western blot analysis of selected components of the PI3K/AKT/mTOR pathway. (**A**) A dose-dependent reduction in the phospho-S6 protein was observed in six chordoma cell lines following a 6 h treatment with buparlisib (0.1 µM and 1 µM). Reduced phospho-AKT levels were seen in U-CH1, MUG-Chor1, UM-Chor1, and MUG-CC1. (**B**) In all four of the chordoma cell lines tested, treatment with rapamycin (1 µM) alone and in combination with palbociclib (0.5 µM) induced a clear reduction in the phospho-S6 protein. No significant effect on phosoho-S6 protein levels was observed following a 6 h incubation with palbociclib alone. The uncropped blots are shown in the Supplementary Materials.

To assess whether the treatments affect the activity of PI3K signaling, Western blot analyses were performed: chordoma cell lines were treated for six hours with buparlisib monotherapy or a combination of palbociclib (0.5 µM) and rapamycin (1 µM). In all six of the cell lines tested, buparlisib reduced the protein levels of phospho-S6-kinase. Phospho-AKT levels decreased in U-CH1, MUG-Chor1, UM-Chor1, and MUG-CC1. Due to the low levels of p-AKT in untreated U-CH2 and U-CH17PII cells, no further reductions were observable following buparlisib treatment. No effect was seen on phospho-ERK (Figure 4A).

In all of the cell lines, Western blot analyses revealed that palbociclib alone did not affect the activity of PI3K signaling; however, treatment with rapamycin reduced the phosphorylation of the mTORC1 downstream target S6 kinase (Figure 4B).

## 4. Discussion

In this study we provide the first evidence for the prognostic relevance of simultaneous PTEN and p16 deficiency in chordoma. We show that both PTEN and p16 negativity are frequent events in chordomas.

In our patient tissue cohort of 43 samples, we observed the loss of PTEN in 28% of the cases, which is in line with the previously published rates of 13% and 26% [8,16]. In one cohort of sacral chordomas PTEN deficiency was postulated to be present in up to 75% [17]. As published previously by von Witzleben et al., 67% of chordomas in our cohort showed p16 negativity, which is in line with former immunohistochemical studies on p16 in chordomas [4,18]. Sommer et al. detected the absence of p16 in 74% of the cases; Cottone et al. reported 53% p16-negative chordomas in their cohort of 303 samples.

So far, there have been no studies dealing with the frequency and prognostic impact of the concomitant deficiency of PTEN and p16 in chordomas. To address this question, we analyzed our cohort and identified a chordoma subgroup of 16% with a combined loss of both tumor suppressor proteins.

Clinicopathological analyses revealed a higher tendency to metastasize and higher KI-67 proliferation rates within this subgroup. Yang et al. recently discussed the clinical relevance of PTEN and p16 as single markers in a cohort of 42 chordomas. A re-evaluation of the published data revealed 28.6% (12/42) with combined PTEN and p16 deficiency. Only one of these twelve cases showed a KI-67 index lower than 5%. Interestingly, six out of seven highly proliferating chordomas (KI-67 > 10%) had a double loss of both markers, further substantiating our results.

Via Kaplan–Meier analyses we observed no significant differences regarding overall survival in patients with the loss of only one protein; however, a tendency towards shorter overall survival emerged. Interestingly, we found that cases of concomitant p16 and PTEN deficiency had a significantly shorter overall survival than patients with only one or no alteration.

The PTEN oncogene has proven but limited prognostic value in chordomas. Yang et al. suggest that PTEN negativity is correlated with a shorter overall and progression-free survival, while Chen et al. only observed an association with shorter PFS in their chordoma cohort of 40 patients [8,17]. Interestingly, copy number loss of the *PTEN* gene locus on chromosomal band 10q23 did not affect overall survival [8].

Various independent studies have assessed p16 as a prognostic biomarker, and in these studies no correlation with clinical outcomes was found. Sommer et al., however, observed an association between strong *CDKN2A* expression and a higher PFS [8,19,20].

Our study indicates that p16 deficiency, as an independent marker, has no prognostic value in chordomas. Although not observed in our cohort, the loss of the PTEN protein might correlate with poor prognoses. Taking into consideration the published frequencies of PTEN negativity and p16 negativity of approximately 20% and 70%, respectively, we assume that a majority (approximately 66%) of PTEN-negative cases are also negative for p16, while p16-negative cases had a concomitant PTEN loss in approximately 25%. Therefore, the prognostic value of PTEN as an independent factor might be biased by the high frequency of PTEN^−^/p16^−^ cases within the PTEN-negative subgroup.

We assume that the combination of both PTEN and p16 as prognostic factors may enhance the prognostic validity; however, as our cohort was limited to a small number of PTEN^−^/p16^−^ cases, results should be re-evaluated in larger or previously published chordoma cohorts.

Based on the frequent loss of p16, the efficacy of palbociclib has been previously investigated in various chordoma cell lines [4]. A phase 2 clinical trial in locally advanced and metastatic chordoma patients has been initiated based on the preclinical results. An interim analysis of the clinical trial revealed stable disease in 33% of patients with manageable toxicity, but no partial or complete response was achieved [21].

Among the main mechanisms of achieved resistance towards CDK4/6 inhibitors are alterations in PI3K/AKT signaling [22]. In various other solid tumors, combination treatments of CDK4/6 inhibitors and inhibitors of PI3K/AKT/mTOR signaling have demonstrated synergistic activities [23,24,25]. A possible explanation for the synergism might be enhanced phosphorylation levels of AKT due to palbociclib treatment, as, for example, observed in breast cancer, osteosarcoma, and glioblastoma cell lines [26,27,28,29].

The frequent loss of PTEN and p16 tumor suppressor proteins provides a rationale for the combined inhibition of both downstream pathways in chordoma. In line with this, we observed synergistic activity for the combination of palbociclib and rapamycin in all six chordoma cell lines.

No synergism was observed for the combination of palbociclib and buparlisib, which might be due to the strong reduction in the cell viability of buparlisib as a single agent. The strong growth inhibitory effects of buparlisib have already been proven in chordoma xenograft models [13].

Notably, we observed no differences in the response to PI3K inhibition between PTEN-negative and PTEN-positive cell lines. This might be explained by the high frequency of mutations in other components of the PI3K/AKT/mTOR pathway, such as PIK3CA, PIK3R1, mTOR, RPS6, and S6, leading to consecutive activity independently of PTEN [9,10,30]. Therefore, co-targeting the CDK4/6 and PI3K/AKT/mTOR signaling pathways might become a novel promising therapeutic strategy for chordoma patients and might be of enhanced value in the subgroup of PTEN^−^/p16^−^ chordomas; however, in order to translate these results towards clinical trials, preclinical evidence should be further substantiated by in vivo studies.

## 5. Conclusions

Here, we first described a novel subgroup of chordoma patients with poor outcomes whose chordomas were simultaneously PTEN- and p16-deficient (PTEN^−^/p16^−^). We observed synergistic effects by simultaneously targeting these frequently altered pathways in chordoma cell lines by using palbociclib and rapamycin. Additionally, the pan-PI3K inhibitor buparlisib dramatically reduced chordoma cell viability and, therefore, may be a promising monotherapy in chordoma patients. Our results provide the first preclinical evidence for the combined inhibition of CDK4/6 as well as PI3K/AKT/mTOR signaling and support the conduction of a clinical trial.

## Figures and Tables

**Table 1:** Clinicopathological features of chordoma cases with respect to their PTEN and p16 statuses. * *p* < 0.05, ** *p* < 0.01, *** *p* < 0.001, derived from the indicated statistical test.

Characteristics	Non-PTEN^−^/p16^−^ (*n* = 36)	PTEN^−^/p16^−^ (*n* = 7)	*p*-Value	PTEN^+^ (*n* = 31)	PTEN^−^ (*n* = 12)	*p*-Value	CDKN2A^+^ (*n* = 14)	CDKN2A^−^ (*n* = 29)	*p*-Value	Statistical Test
** Gender **										Chi-squared test
Male (n)	23	5	0.65	20	8	0.89	10	18	0.55	
Female (n)	13	2		11	4		4	11		
** Age ** (Years; mean ± SD)	61.64 ± 18.14	57.86 ± 17.81	0.61	64.29 ± 16.38	52.58 ± 17.08	* 0.049	57.36 ± 19.15	62.79 ± 16.18	0.35	Student’s *t*-test
** Ki-67 **										Chi-squared test
<10%	30	3	* 0.04	27	7	* 0.038	12	22	0.46	
≥10%	6	4		4	5		2	7		
** Maximal Diameter ** (cm ± SD)	7.13 ± 8.86	10 ± 3.71	0.42	6.01 ± 8.96	11.19 ± 4.5	0.072	10.97 ± 12.5	5.76 ± 4.45	0.058	Student’s *t*-test
** Recurrence **										Chi-squared test
Presence (n)	18	5	0.26	14	9	0.078	9	14	0.21	
Absence (n)	18	2		17	3		4	15		
** Metastasis **										Chi-squared test
Presence (n)	6	5	** 0.0026	3	8	*** 0.00012	4	7	0.75	
Absence (n)	30	2		28	4		10	22		
** Location **										Fisher’s exact test
Clivus (n)	6	0	0.47	6	0	0.21	3	3	0.25	
Mobile spine (n)	10	1		9	2		4	7		
Os sacrum (n)	19	6		15	10		6	19		
Other (n)	1	0		1	0		1	0		

## Data Availability

The data presented in this study are available in this article (and supplementary material).

## Supplementary Materials

The following supporting information can be downloaded at: https://www.mdpi.com/article/10.3390/cancers15071977/s1, Figure S1: Representative inhibition curves of Sirolimus and Palbociclib single treatment in six chordoma cell lines.
