# Supplementary material for: Prognostic Relevance and In Vitro Targeting of Concomitant PTEN and p16 Deficiency in Chordomas
Source: Cancers (Basel). 2023 Mar 26;15(7):1977. doi: 10.3390/cancers15071977 (PMC10093147; doi:10.3390/cancers15071977)
Supplement: Supplementary file 1 [file cancers-15-01977-s001.zip › cancers-2290869-supplementary.pdf]

Supplementary Material

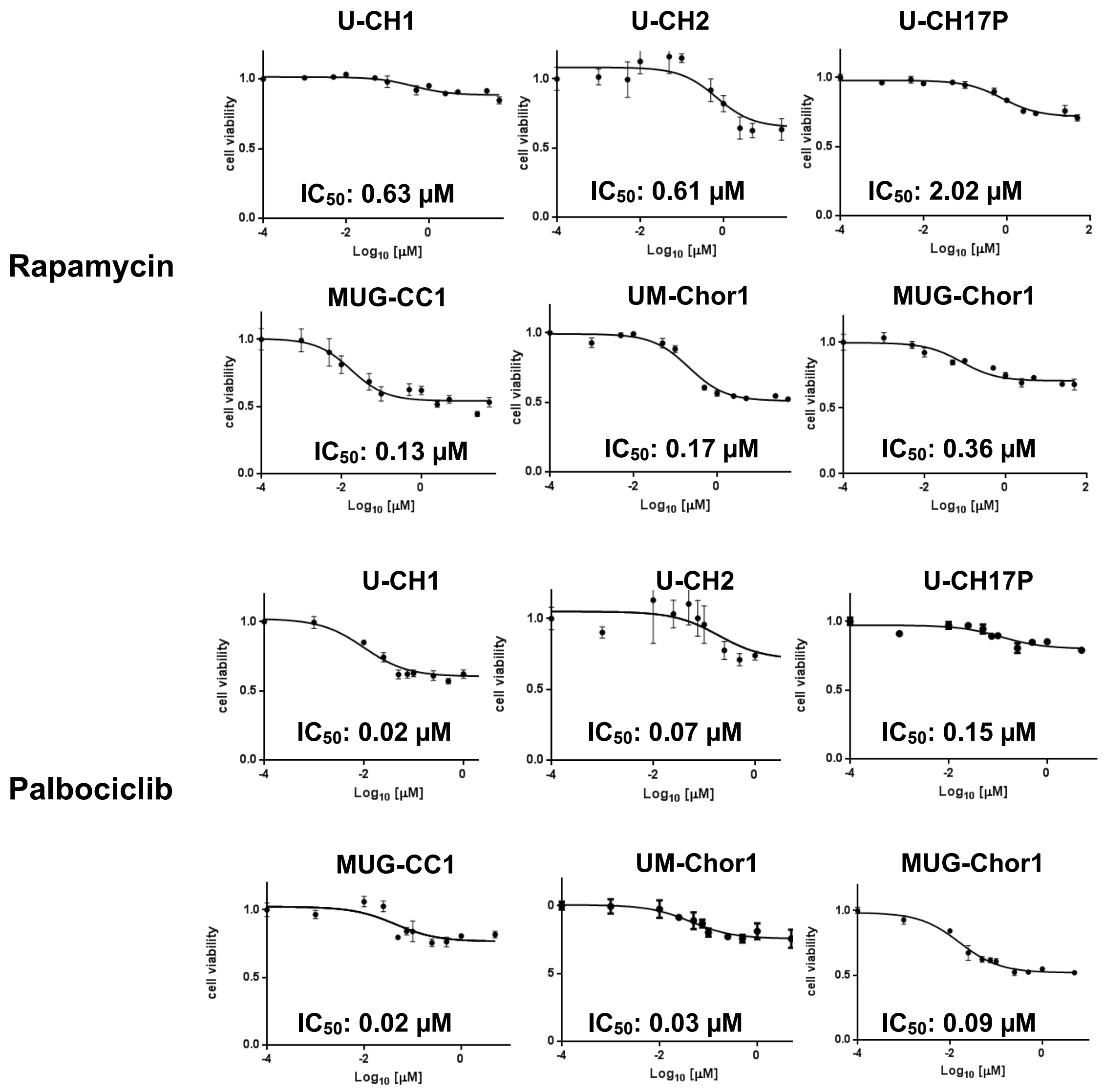

Supplement Figure S1: Representative inhibition curves of Sirolimus and Palbociclib single treatment in six chordoma cell lines. The mean IC50 values of biological triplicates is given for each cell line.

Uncropped Western blots

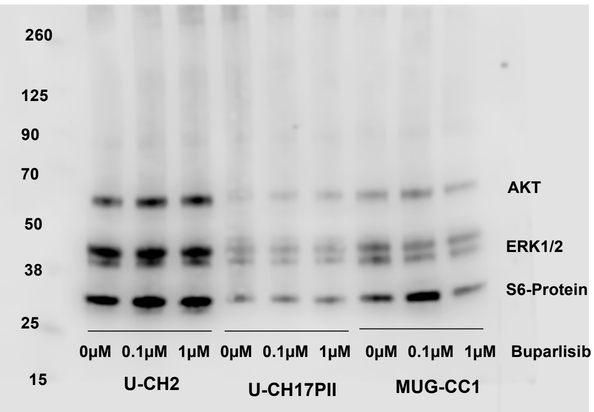

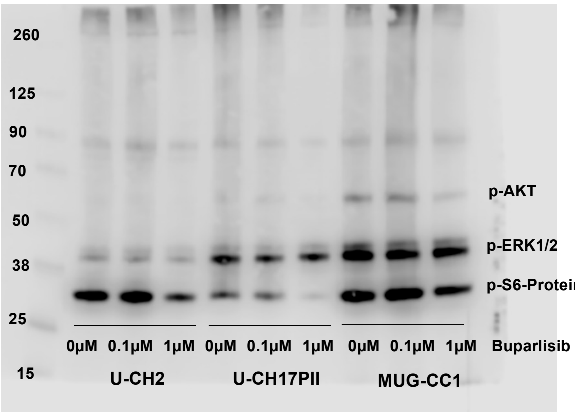

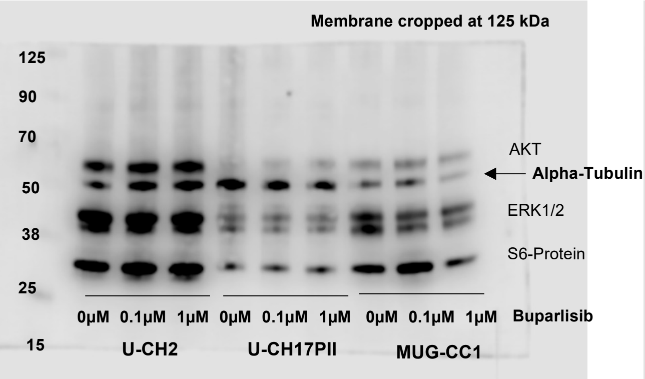

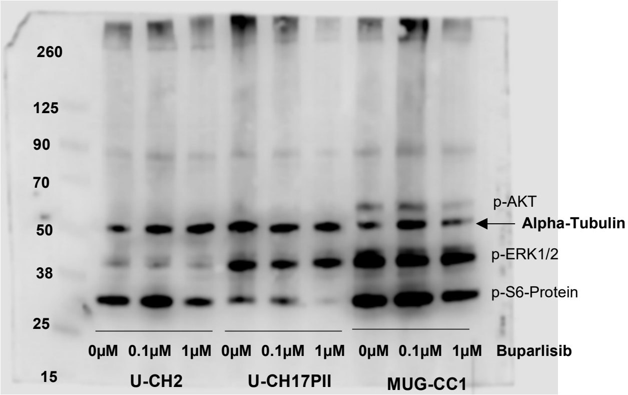

Signal intensities measured using ImageJ:

	U-CH2			U-CH17PII			MUG-CC1		
**Buparlisib conc.**	**0 µM**	**0.1 µM**	**1 µM**	**0 µM**	**0.1 µM**	**1 µM**	**0 µM**	**0.1 µM**	**1 µM**
AKT	78300	114000	116000	7860	18100	9920	37500	52100	23600
ERK1/2	201000	214000	198000	54000	49600	48700	117000	90900	67400
S6 protein	153000	236000	197000	38000	42600	42200	79900	184000	48100
alpha-Tubulin	14100	35300	39000	46400	31600	23500	11700	14200	5300

	U-CH2			U-CH17PII			MUG-CC1		
**Buparlisib conc.**	**0 µM**	**0.1 µM**	**1 µM**	**0 µM**	**0.1 µM**	**1 µM**	**0 µM**	**0.1 µM**	**1 µM**
p-AKT	3870	1390	1440	8540	3300	1320	21800	22100	7410
p-ERK1/2	23800	25400	19000	71500	54400	65200	143000	112000	105000
p-S6 protein	116000	143000	47300	47700	32500	4830	143000	198000	82900
alpha-Tubulin	13500	28800	40800	42100	32200	30100	12600	33600	11700

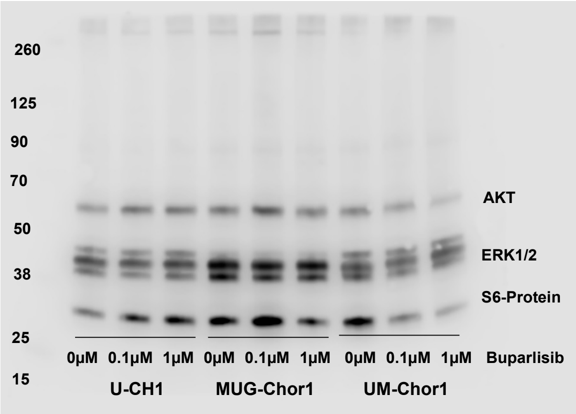

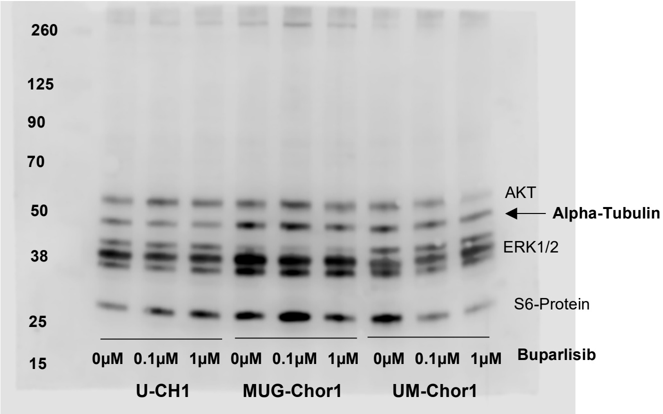

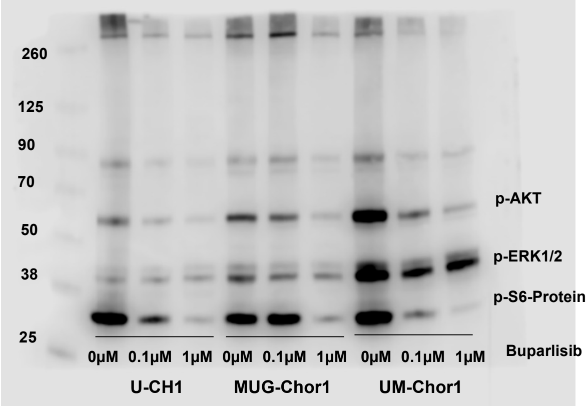

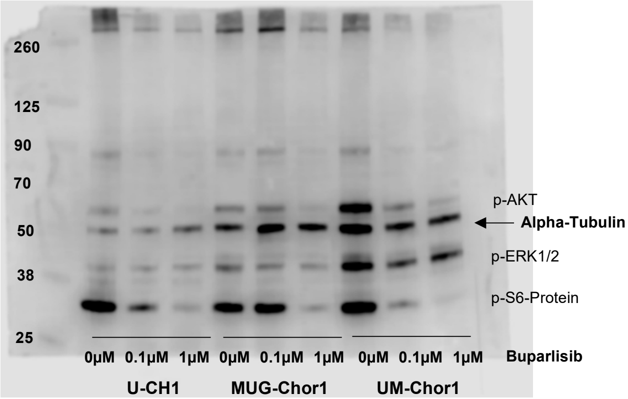

Signal intensities measured using ImageJ:

	U-CH1			MUG-Chor1			UM-Chor1		
**Buparlisib conc.**	**0 µM**	**0.1 µM**	**1 µM**	**0 µM**	**0.1 µM**	**1 µM**	**0 µM**	**0.1 µM**	**1 µM**
AKT	61700	116000	112000	86400	151000	69800	97000	72000	37700
ERK1/2	335000	302000	357000	513000	447000	446000	365000	301000	302000
S6 protein	99500	155000	195000	231000	348000	144000	250000	101000	54400
alpha-Tubulin	39500	30500	26900	64500	72500	43800	54200	46400	41200

	U-CH1			MUG-Chor1			UM-Chor1		
**Buparlisib conc.**	**0 µM**	**0.1 µM**	**1 µM**	**0 µM**	**0.1 µM**	**1 µM**	**0 µM**	**0.1 µM**	**1 µM**
p-AKT	43200	11800	5930	52700	28500	11900	196000	38800	19300
p-ERK1/2	42900	25800	22600	72700	50400	41700	254000	115000	144000
p-S6 protein	227000	51300	14800	139000	159000	19700	275000	36600	6580
alpha-Tubulin	10700	9910	19800	28800	60900	39100	97500	38200	38200

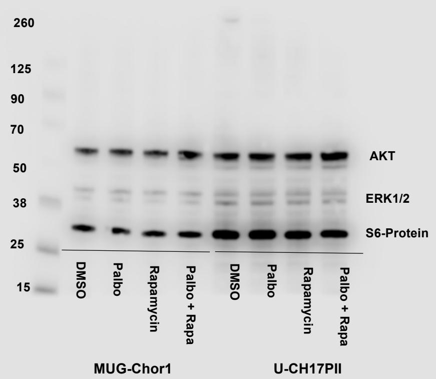

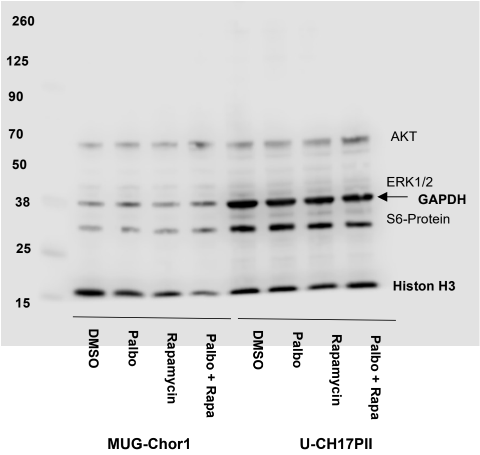

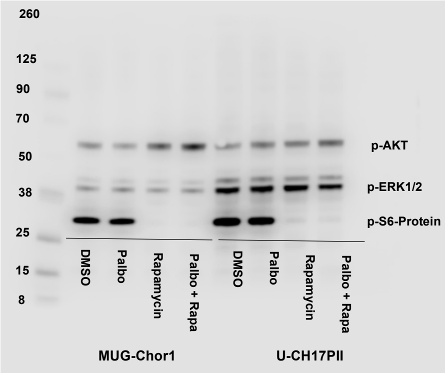

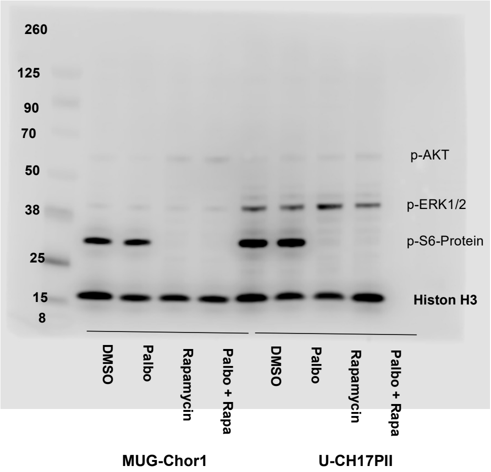

Signal intensities measured using ImageJ:

	U-CH1				UM-Chor1			
	DMSO	Palbo	Rapamycin	Palbo + Rapa.	DMSO	Palbo	Rapamycin	Palbo + Rapa.
AKT	27300	24900	40200	16100	29500	43600	36900	23500
ERK1/2	43800	61900	79700	46600	78600	134000	180000	104000
S6 protein	36400	24800	54800	14600	32200	49500	47500	18100
Histon H3	26400	38500	60400	52900	53300	75900	78500	46100

	U-CH1				UM-Chor1			
	DMSO	Palbo	Rapamycin	Palbo + Rapa.	DMSO	Palbo	Rapamycin	Palbo + Rapa.
p-AKT	14400	12100	9340	12900	97500	123000	93400	70500
p-ERK1/2	7780	13700	13100	6870	108000	168000	139000	77700
p-S6 protein	24800	13900	-1080	-766	68000	115000	15700	702
Histon H3	32900	56500	61100	42200	89200	128000	75300	45700

	MUG-Chor1				U-CH17PII			
	DMSO	Palbo	Rapamycin	Palbo + Rapa.	DMSO	Palbo	Rapamycin	Palbo + Rapa.
AKT	55300	65000	65100	103000	91200	102000	104000	144000
ERK1/2	15200	20300	17700	18700	44400	44000	43900	33100
S6 protein	73400	57500	75700	89500	223000	248000	202000	185000
Histon H3	247000	175000	141000	78400	209000	189000	162000	231000

	MUG-Chor1				U-CH17PII			
	DMSO	Palbo	Rapamycin	Palbo + Rapa.	DMSO	Palbo	Rapamycin	Palbo + Rapa.
p-AKT	55900	42300	91800	129000	37900	82800	55600	97900
p-ERK1/2	50100	52500	40500	46500	216000	209000	228000	174000
p-S6 protein	257000	214000	2930	4600	281000	313000	19300	6780
Histon H3	148000	86700	83200	85600	134000	87700	80500	143000
